# Supplementary material for: Associations of Maternal Consumption of Dairy Products during Pregnancy with Perinatal Fatty Acids Profile in the EDEN Cohort Study
Source: Nutrients. 2022 Apr 14;14(8):1636. doi: 10.3390/nu14081636 (PMC9025886; doi:10.3390/nu14081636)
Supplement: Supplementary file 1 [file nutrients-14-01636-s001.zip › nutrients-1663580-supplementary.pdf]

**Table S1.** Comparison of included and non-included participants characteristic[1]

| | Maternal blood | | | | | Cord blood | | | | | Colostrum | | | | |
|---|---|---|---|---|---|---|---|---|---|---|---|---|---|---|---|
| | *n* | **Included** | *n* | **Non-included** | *p*[2] | *n* | **Included** | *n* | **Non-included** | *p*[2] | *n* | **Included** | *n* | **Non-included** | *p*[2] |
| Poitiers, % | 1754 | 50 (869) | 248 | 40 (99) | 0.004 | 1337 | 53 (708) | 665 | 39 (260) | <0.0002 | 1123 | 34 (299) | 879 | 60 (669) | <0.0002 |
| Age at delivery (years) | 1754 | 29 (5) | 153 | 29 (5) | 0.67 | 1337 | 29 (5) | 570 | 29 (5) | 0.71 | 1028 | 30 (5) | 879 | 29 (5) | 0.23 |
| Educational attainment, % | 1741 | | 169 | | 0.001 | 1326 | | 584 | | 0.02 | 1038 | | 872 | | <0.0001 |
| <high school diploma | | 27 (478) | | 42 (71) | | | 27 (357) | | 33 (192) | | | 22 (189) | | 35 (360) | |
| high school diploma | | 18 (314) | | 15 (26) | | | 17 (232) | | 18 (108) | | | 15 (135) | | 20 (205) | |
| 2-year university degree | | 22 (384) | | 18 (30) | | | 23 (305) | | 19 (109) | | | 25 (214) | | 19 (200) | |
| >2-year university degree | | 32 (565) | | 25 (42) | | | 33 (432) | | 30 (175) | | | 38 (334) | | 26 (273) | |
| Monthly household income, % | 1744 | | 169 | | 0.002 | 1327 | | 586 | | 0.90 | 1040 | | 873 | | <0.0001 |
| <1500 € | | 16 (287) | | 24 (40) | | | 17 (225) | | 17 (102) | | | 13 (111) | | 21 (216) | |
| 1500-2300 € | | 29 (506) | | 37 (62) | | | 29 (390) | | 30 (178) | | | 27 (240) | | 32 (328) | |
| 2301-3000 € | | 27 (466) | | 21 (35) | | | 27 (354) | | 25 (147) | | | 27 (237) | | 25 (264) | |
| >3000 € | | 28 (485) | | 19 (32) | | | 27 (358) | | 27 (159) | | | 33 (285) | | 22 (232) | |
| BMI before pregnancy (kg/m$^2$), % | 1727 | | 157 | | 0.44 | 1310 | | 574 | | 0.05 | 1018 | | 866 | | <0.0001 |
| <18.5 | | 8 (146) | | 10 (15) | | | 9 (120) | | 7 (41) | | | 10 (83) | | 8 (78) | |
| 18.5-24.9 | | 66 (1134) | | 59 (93) | | | 66 (866) | | 63 (361) | | | 70 (602) | | 61 (625) | |
| 25.0-29.9.0 | | 17 (298) | | 20 (32) | | | 17 (221) | | 19 (109) | | | 14 (120) | | 21 (210) | |
| ≥30.0 | | 9 (149) | | 11 (17) | | | 8 (103) | | 11 (63) | | | 7 (61) | | 10 (105) | |
| Gestational diabetes, % | 1753 | 6 (110) | 151 | 9 (13) | 0.26 | 1336 | 6 (85) | 568 | 7 (38) | 0.79 | 1026 | 6 (49) | 878 | 7 (74) | 0.15 |
| Hypertensive disorders during pregnancy, % | 1753 | | 151 | | 0.96 | 1336 | | 568 | | 0.47 | 1026 | | 878 | | 0.07 |
| Gestational hypertension | | 3 (51) | | 3 (5) | | | 3 (36) | | 4 (20) | | | 3 (30) | | 3 (26) | |
| Preeclampsia | | 2 (37) | | 2 (3) | | | 2 (26) | | 2 (14) | | | 1 (12) | | 3 (28) | |
| Smoking during pregnancy, % | 1712 | 26 (450) | 135 | 25 (34) | 0.78 | 1304 | 27 (351) | 543 | 24 (133) | 0.28 | 983 | 23 (199) | 864 | 29 (285) | 0.004 |
| Any breastfeeding duration (months), % | 1749 | | 142 | | 0.80 | | | | | | 1016 | | 875 | | <0.0001 |
| Never | | 27 (477) | | 26 (37) | | | 29 (384) | 557 | 23 (130) | 0.12 | | 0 (1) | | 50 (513) | |

| | | | | | | |
|---|---|---|---|---|---|---|
| < 3.0 | 27 (464) | 27 (38) | 26 (346) | 28 (156) | 34 (296) | 20 (206) |
| 3.0-5.9 | 23 (398) | 26 (37) | 23 (301) | 24 (134) | 32 (284) | 15 (151) |
| ≥ 6.0 | 23 (410) | 21 (30) | 23 (303) | 25 (137) | 34 (294) | 14 (146) |

[1] Values are the mean ± SD or % (*n*). [2] Student's t-test and Chi-square. Significance level was set at alpha=0.05.

**Table S2.** Description of maternal DP consumption in the study populations [1]

| | EDEN full cohort ($n$ = 1782) | Study populations | | |
|---|---|---|---|---|
| | | Maternal blood ($n$ = 1754) | Cord blood ($n$ = 1337) | Colostrum ($n$ = 879) |
| **DP consumption tertiles during pregnancy** | | | | |
| Low-consumer, g/d | | | | |
| Higher-fat content DP | 23 ± 11 | 24 ± 11 | 24 ± 11 | 25 ± 10 |
| Lower-fat content DP | 169 ± 89 | 169 ± 89 | 165 ± 91 | 173 ± 87 |
| Moderate consumer, g/d | | | | |
| Higher-fat content DP | 58 ± 11 | 58 ± 11 | 58 ± 11 | 59 ± 11 |
| Lower-fat content DP | 431 ± 76 | 431 ± 76 | 430 ± 76 | 430 ± 75 |
| Heavy consumer, g/d | | | | |
| Higher-fat content DP | 153 ± 86 | 153 ± 86 | 155 ± 86 | 151 ± 81 |
| Lower-fat content DP | 815 ± 229 | 814 ± 230 | 829 ± 236 | 806 ± 215 |

[1] Values are the mean ± SD.

**Table S3.** Description of maternal RBC membrane, cord RBC membrane and colostrum fatty acids [1].

| | Maternal blood ($n$ = 1754) | Cord blood ($n$ = 1337) | Colostrum ($n$ = 879) |
|---|---|---|---|
| C15:0, % total fat | 0.17 ± 0.03 | 0.12 ± 0.03 | 0.42 ± 0.07 |
| C17:0, % total fat | 0.31 ± 0.04 | 0.32 ± 0.08 | 0.27 ± 0.05 |
| C18:3 n-3 (ALA), % total fat | 0.10 ± 0.03 | 0.05 ± 0.03 | 0.65 ± 0.21 |
| C20:6 n-3 (DHA), % total fat | 5.32 ± 0.94 | 6.21 ± 0.91 | 0.64 ± 0.19 |
| n-3 PUFAs, % total fat | 7.80 ± 1.20 | 7.06 ± 1.06 | 2.05 ± 0.41 |
| C18:2 n-6 (LA), % total fat | 7.15 ± 0.81 | 2.89 ± 0.44 | 9.83 ± 1.83 |
| C20:4 n-6 (AA), % total fat | 12.87 ± 0.82 | 15.30 ± 0.90 | 0.86 ± 0.16 |
| n-6 PUFAs, % total fat | 25.63 ± 1.35 | 26.23 ± 1.11 | 12.18 ± 1.89 |
| n-6:n-3 LC-PUFA ratio | 2.5 ± 0.5 | 3.4 ± 0.6 | 1.8 ± 0.4 |

[1] Values are the mean ± SD.

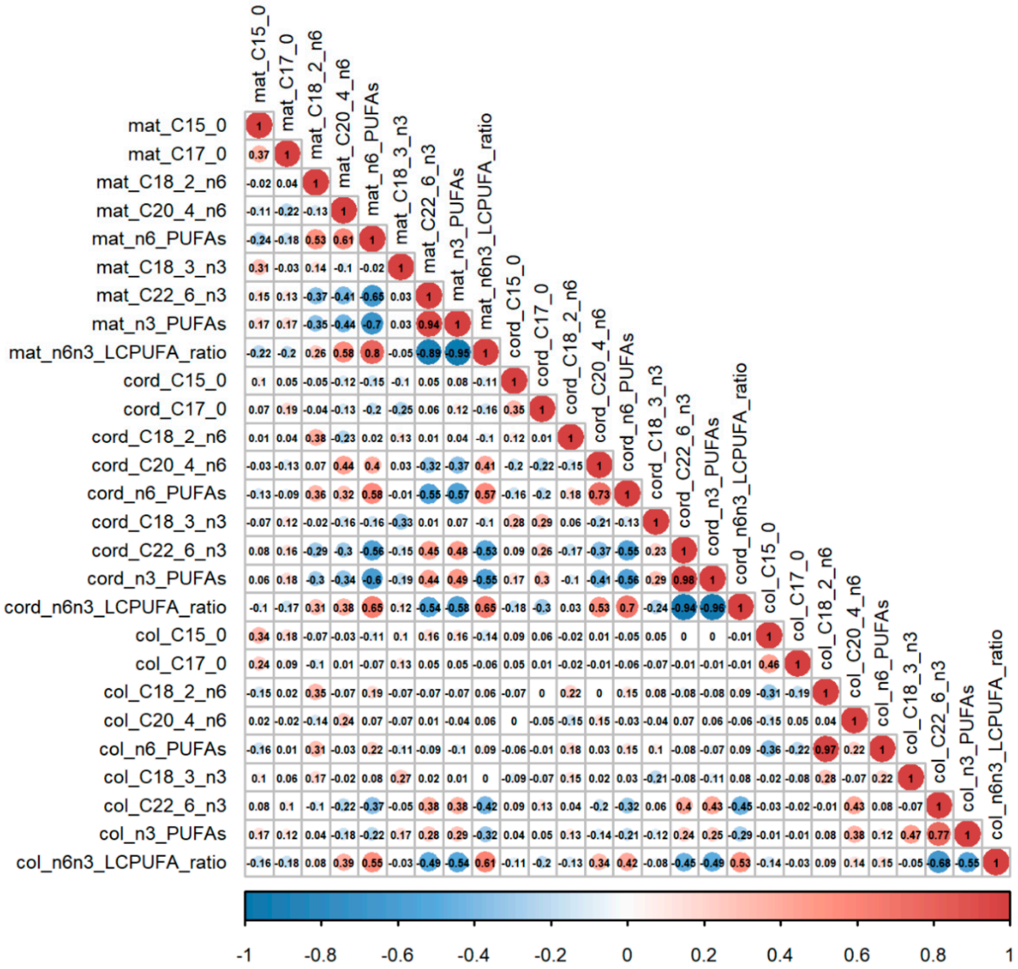

**Figure S1.** Heatmap of the Pearson's correlation between perinatal biofluids fatty acids concentration. Prefixes "mat_" ,"cord_", and "col" indicate maternal RBC membrane, cord RBC membrane and colostrum, respectively. Numbers in the cells are Pearson's correlation coefficient *r*. The size of the circle inside each cell represent the magnitude of the correlation.

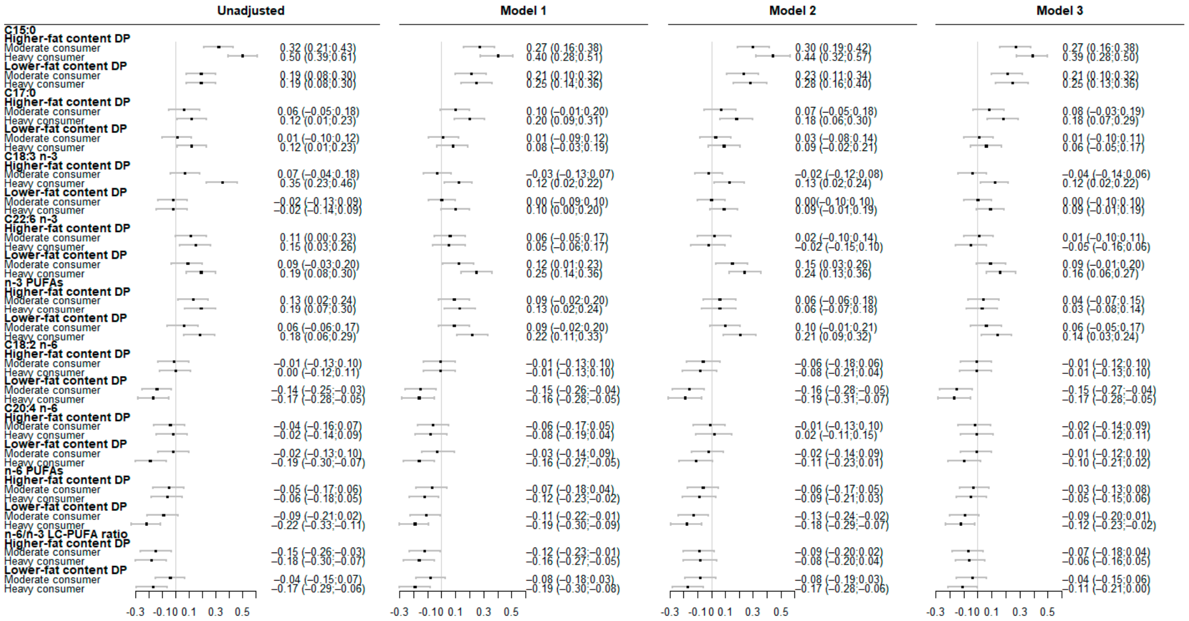

**Figure S2.** Associations between maternal higher-fat and lower-fat content DP consumption tertiles and fatty acids levels in maternal RBC membrane (β (95%CI)). Results are from linear regression models. Model 1 was adjusted on study center and maternal age at delivery. Model 2 was adjusted on model 1 covariates and on maternal "healthy" dietary pattern during pregnancy. Model 3 was adjusted on model 1 covariates and on the frequency of fish consumption during pregnancy. Higher-fat and lower-fat content DP consumption tertiles were studied simultaneously within each model.

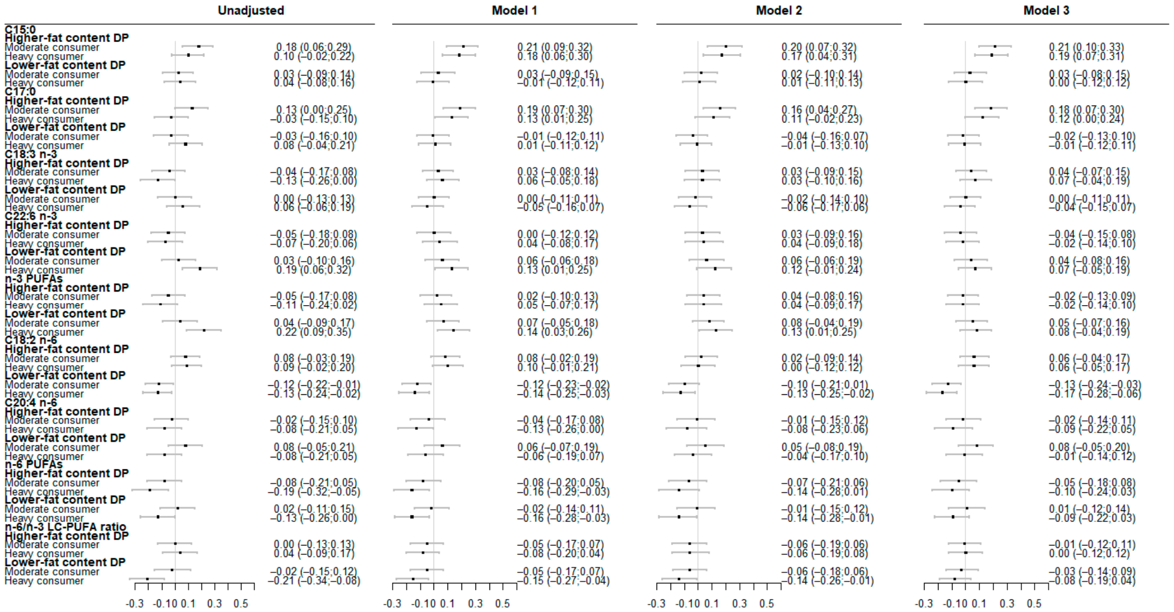

**Figure S3.** Associations between maternal higher-fat and lower-fat content DP consumption tertiles and fatty acids levels in cord RBC membrane (β (95%CI)).Results are from linear regression models. Model 1 was adjusted on study center and maternal age at delivery. Model 2 was adjusted on model 1 covariates and on maternal "healthy" dietary pattern during pregnancy. Model 3 was adjusted on model 1 covariates and on the frequency of fish consumption during pregnancy. Higher-fat and lower-fat content DP consumption tertiles were studied simultaneously within each model.

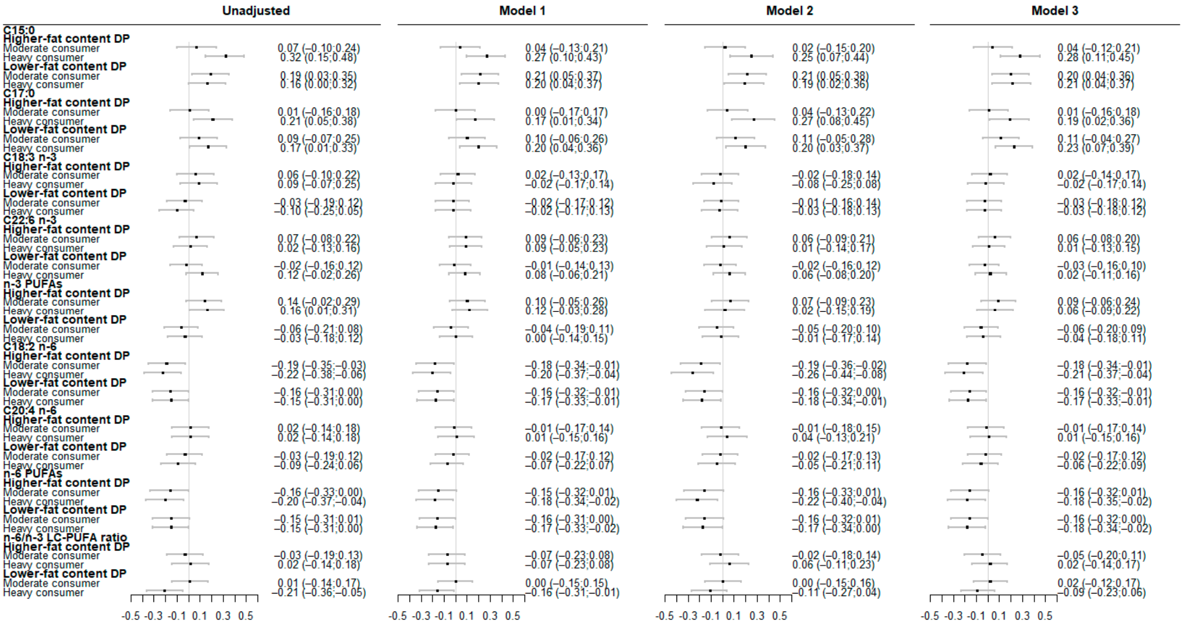

**Figure S4.** Associations between maternal higher-fat and lower-fat content DP consumption tertiles and fatty acids levels in colostrum (β (95%CI)). Results are from linear regression models. Model 1 was adjusted on study center and maternal age at delivery. Model 2 was adjusted on model 1 covariates and on maternal “healthy” dietary pattern during pregnancy. Model 3 was adjusted on model 1 covariates and on the frequency of fish consumption during pregnancy. Higher-fat and lower-fat content DP consumption tertiles were studied simultaneously within each model.
